# Leukaemia Stem Cells and Their Normal Stem Cell Counterparts Are Morphologically Distinguishable by Artificial Intelligence

**DOI:** 10.1111/jcmm.70564

**Published:** 2025-05-19

**Authors:** Dongguang Li, Ngoc DeSouza, Kathy Nguyen, Shaoguang Li

**Affiliations:** ^1^ Division of Hematology/Oncology, Department of Medicine University of Massachusetts Chan Medical School Worcester Massachusetts USA

**Keywords:** artificial intelligence, leukaemia stem cells, morphology

## Abstract

Leukaemia stem cells (LSCs) are a rare population among the bulk of leukaemia cells and are responsible for disease initiation, progression/relapse and insensitivity to therapies in numerous haematologic malignancies. Identification of LSCs and monitoring of their quantity before, during, and after treatments will provide a guidance for choosing a correct treatment and assessing therapy response and disease prognosis, but such a method is still lacking simply because there are no distinct morphological features recognisable for distinguishing LSCs from normal stem cell counterparts. Using artificial intelligence (AI) deep learning and polycythemia vera (PV) as a disease model (a type of human myeloproliferative neoplasms derived from a haematopoietic stem cell harbouring the JAK2V617F oncogene), we combine 19 convolutional neural networks as a whole to build AI models for analysing single‐cell images, allowing for distinguishing between LSCs from JAK2V617F knock‐in mice and normal stem counterparts from healthy mice with a high accuracy (> 99%). We prove the concept that LSCs possess unique morphological features compared to their normal stem cell counterparts, and AI, but not microscopic visualisation by pathologists, can extract and identify these features. In addition, we show that LSCs and other cell lineages in PV mice are also distinguishable by AI. Our study opens up a potential AI morphology field for identifying various primitive leukaemia cells, especially including LSCs, to help assess therapy responses and disease prognosis in the future.

## 1 Introduction

It is generally believed that LSCs are closely related to disease initiation/progression, relapse and drug resistance/insensitivity in many types of haematologic malignancies [1, 2, 3]. An unmet need is to obtain an ability to monitor and quantify LSCs for proposing correct treatment, evaluating treatment response and predicting disease outcomes, but a method that specifically identifies this stem cell population is still lacking in medical practice. Although some cell surface markers are used for detecting stem cells by flow cytometry and other means, those methods do not distinguish LSCs from their normal cell counterparts because both cancer and normal stem cells often share and express similar markers [4, 5]. Furthermore, analysis of pathological cell and tissue images is a gold standard for characterisation and diagnosis of human diseases, but morphological differences between LSCs and their normal stem cell counterparts have not been recognisable by human visualisation, although we believe that these differences exist. A novel method needs to be developed to specifically identify LSCs and distinguish them from their normal stem cell counterparts, as well as other cancer and normal cell populations in the same patients. Based on our recent advances in using artificial intelligence (AI) to read cell or tissue images for disease diagnosis [6, 7], we believe that AI deep learning will allow extraction and identification of the morphological features presented by LSCs, normal stem cells and other related cell populations. Because polycythemia vera (PV) is a major form of human myeloproliferative neoplasms (MPNs) that are derived from LSCs and still not curable with available therapies, here we use PV as a model disease and AI deep learning to prove the concept that cancer and normal stem cells are morphologically distinguishable, which would allow us to propose a novel idea that acquisition of genetic lesions causes transformation of a normal stem cell into a LSC with morphological changes recognisable by AI but not by human microscopic visualisation. Our study provides the first example of identifying LSCs and distinguishing them from their normal stem cell counterparts by viewing pathologic cell images using AI deep learning.

## 2 Methods

### 2.1 Mice

Conditional JAK2V617F knock‐in mice in C57BL/6 background were kindly provided by Dr. Golam Mohi at the University of Virginia, Charlottesville, Virginia, USA. These mice develop myeloproliferative disease [8, 9]. Wild‐type C57BL/6 littermates were produced during the breeding of JAK2V617F knock‐in mice.

### 2.2 Single‐Cell Image Preparation

Bone marrow cells from JAK2V617F knock‐in mice were flushed out and stained with various antibodies for sorting by flow cytometry. Various primitive bone marrow cells were sorted using the following antibodies: Lin^−^Sca‐1^+^c‐Kit^+^CD150^+^CD48^−^ for long‐term haematopoietic stem cells (LT‐HSCs), Lin^−^Sca‐1^+^c‐Kit^+^CD150^−^CD48^−^ for short‐term haematopoietic stem cells (ST‐HSCs) and Lin^−^Sca‐1^+^c‐Kit^+^CD150^−^CD48^+^ for multipotent progenitors (MPPs). The antibody cocktail that allows elimination of mature cells to purify lineage‐negative cells includes CD3, CD4, CD8, B220, Gr‐1, Mac‐1 and Ter119. The sorted cells were spun onto coverslips for staining with Giemsa and Wright (G&W). The stained cells were scanned to form photoshop files, and single‐cell images were prepared by cropping. The single‐cell colour images were converted to grayscale images. In brief detail, average pixel values (ranging from 0 to 255) of the primary colours, which are red, green and blue (popularly referred to as RGB) are combined. The luminous intensity of each colour band (which is 24 bits) is combined into a reasonable approximated grayscale value (8 bits). The RGB values are converted to grayscale (returns the monochrome luminance of the given colour as an intensity between 0 and 255) using the NTSC formula: 0.299 × Red + 0.587 × Green + 0.114 × Blue. This formula closely represents the average person's relative perception of the brightness of red, green and blue light. NTSC is an abbreviation for National Television Standards Committee, named for the group that originally developed the black & white and subsequently colour television system. Next, the grayscale images were processed by contrast enhancement functions to form image datastore for further analysis by AI.

### 2.3 Algorithms and Training Options

The initialisation of the deep learning model was random, thereby producing some differences between model trainings. Therefore, a neural network was fine‐tuned to reach the highest accuracy. Each of the 19‐CNNs was fine‐tuned in training before combining them into one system. A set of options was created for training these networks using three different optimizers: stochastic gradient descent with momentum (SGDM), adaptive moment estimation (ADAM) and root mean square propagation (RMSProp). The learning rate varied from 0.001 to 0.0001. The maximum number of epochs for training was between 20 and100, and a mini batch with 32–64 observations was used at each iteration. The training data split ratio of training:validating:testing was set to 0.6:0.2:0.2.

### 2.4 Transfer Learning

We took advantage of available model architectures and fine‐tuned deeper layers in the neural network by training it on our new dataset in order to learn features specific to our new dataset. We applied transfer learning on the ImageNet dataset, which consists of natural images, with large numbers of more than 14 million images distributed over 1000 classes, including images for objects, animals and humans. All selected pretrained models were initialised with ImageNet pretrained weights and biases.

### 2.5 Selection of Pretrained Convolutional Neural Networks

We selected 19 pretrained image classification neural networks that had already learned to extract powerful and informative features from being trained on a subset of the ImageNet database, which is used in the ImageNet Large‐Scale Visual Recognition Challenge (ILSVRC), because these neural networks had been trained on more than a million images and could classify images into 1000 object categories.

### 2.6 Hardware and Software

The Matlab Deep Learning Toolbox provided a framework for designing and implementing deep neural networks with algorithms, pretrained models and apps. Image Processing Toolbox provided a comprehensive set of reference‐standard algorithms and workflow apps for image processing, analysis, visualisation and algorithm development. The networks in this study were all trained and tested using Matlab software (MATLAB R2021a) and an NVidia Titan XP GPU.

### 2.7 Code and Data Availability

The source code including the deep learning models developed in this study and the datasets used can be downloaded from our FTP site at https://fts.umassmed.edu (user name: lid1; password: April2000april2000) in the folder of/Home/lid1/Stem Cell Data or from the corresponding author upon reasonable request to shaoguang.li@umassmed.edu.

## 3 Results

### 3.1 JAK2V617F Causes Morphological Changes of LSCs in PV

**FIGURE 1:**
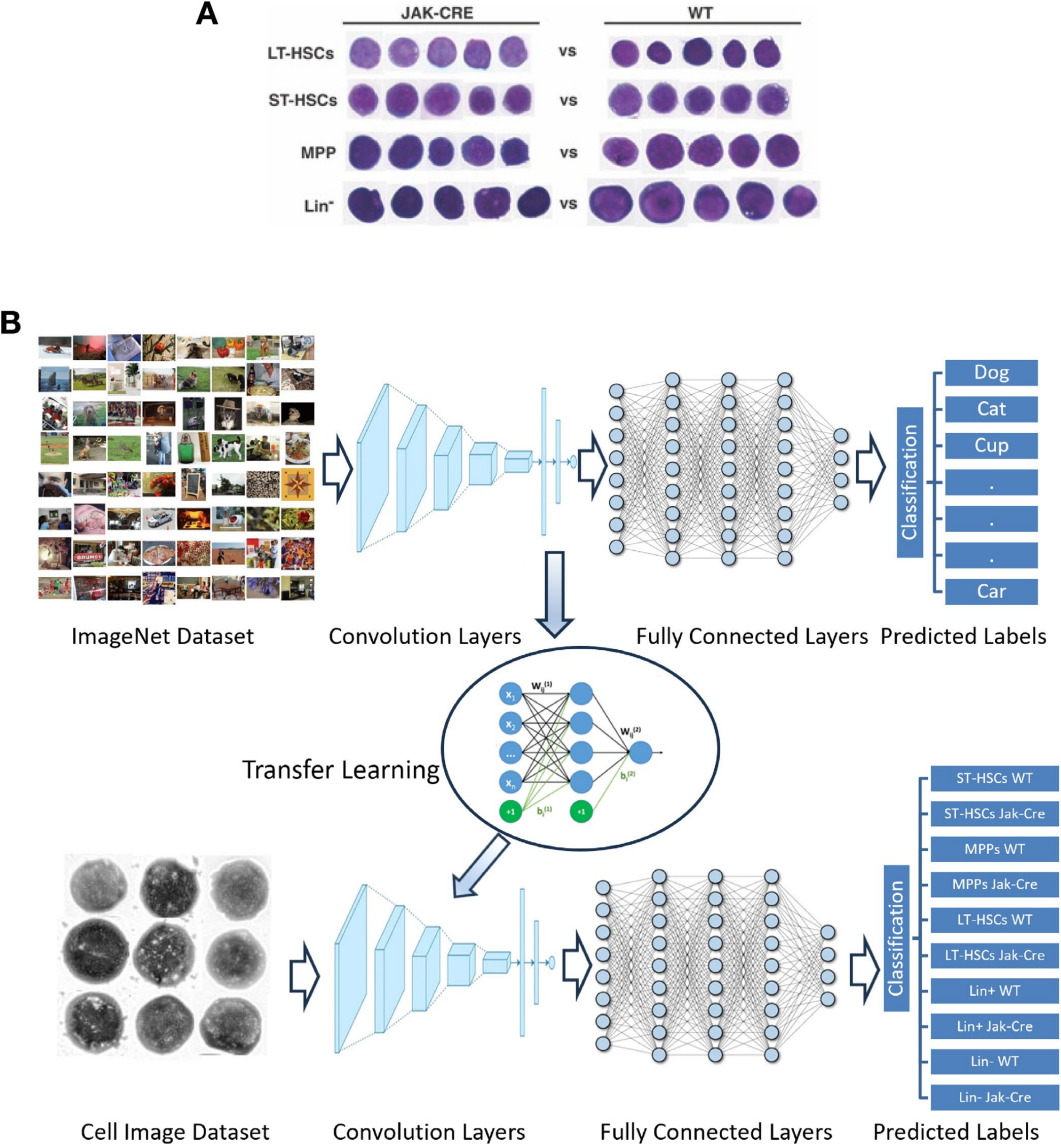
Transfer learning strategy in building deep learning models. (A) Microscopic visualisation of G&W‐stained LSK cells and Lin^−^ (Lineage‐negative) by human eyes could not show distinguishable morphological features among JAK2V617F‐expressing and non‐JAK2V617F‐expressing LSK cells (LT‐HSCs, ST‐HSCs and MPPs) and Lin^−^ cells from mice. (B) Deeper layers in the pretrained CNNs were further fine‐tuned on our new stem cell dataset to learn feature specific to our new dataset.

In PV, HSCs acquire the JAK2V617F mutation to become LSCs [10]. We hypothesised that the acquisition of JAK2V617F not only transforms normal HSCs to LSCs but also causes morphological changes of the cells, providing the conceptual basis for morphologically distinguishing LSCs from their normal stem cell counterparts. In mice, the HSCs reside in the LSK (Lin^−^Sca‐1^+^c‐Kit^+^) cell population in bone marrow, and LSK cells are composed of three populations: LT‐HSCs, ST‐HSCs and MPPs. To test our hypothesis, we obtained JAK2V617F‐expressing LT‐HSCs, ST‐HSCs and MPPs from the bone marrow of JAK2V617F knock‐in mice (Jak‐Cre) [8], and used normal LT‐HSCs, ST‐HSCs and MPPs from the bone marrow of wild type (WT) mice as controls. Through our microscopic visualisation of G&W‐stained LSK cells, we did not observe distinguishable morphological features among JAK2V617F‐expressing and non‐JAK2V617F‐expressing LT‐HSCs, ST‐HSCs, MPPs and Lin^−^ (lineage‐negative) cells (Figure 1A), but we believed that JAK2V617F‐caused morphological changes of LSCs in PV could be extracted and identified by AI deep learning, although a difficult technical challenge would be expected. Keeping this technical difficulty in mind, for achieving high accuracy of recognising LSCs in PV, we felt that we needed to apply transfer learning creatively in building our deep learning models to allow for a faster, easier training of a neural network using a relatively small dataset for the current task. Our transfer learning strategy was that we pretrained a subset of the ImageNet database containing millions of images that can be classified into 1000 object categories [6], which has been used in the ImageNet Large‐Scale Visual Recognition Challenge (ILSVRC). This approach enables us to avoid training a neural network from scratch. Following this strategy, we pretrained CNN models on the ImageNet dataset that contains more than 14 million images distributed over 1000 classes, such as objects, animals and humans for face detection, distinguishing types of animals, types of flowers, etc. We then fine‐tuned deeper layers in the pretrained CNNs for further training on our new stem cell dataset and learned features specific to our new dataset (Figure 1B).

**FIGURE 2:**
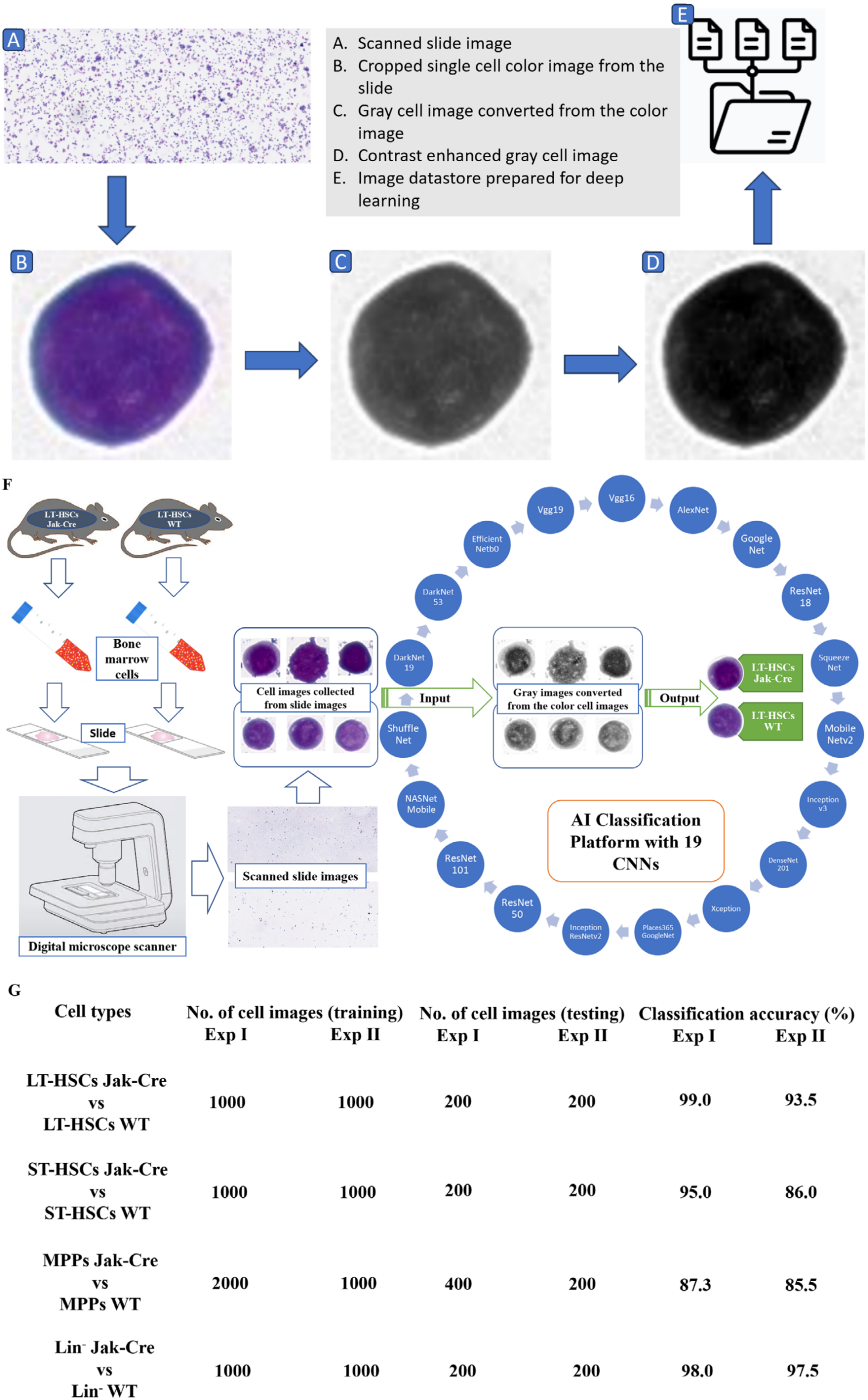
Establishment of AI models and analysis of single‐cell images for distinguishing LSCs from normal stem cell counterparts. (A–E) Workflow of AI the system. Photographs of the slide images and scanned images of the entire slides for DLBCL and non‐DLBCL were used for establishing AI models. (F) Overview of data flow implemented and components of the GOTDP‐MP‐CNNs. 17 CNNs [AlexNet, GoogleNet (ImageNet), GoogleNet (Places365), ResNet18, ResNet50, ResNet101, Vgg16, Vgg19, Inceptionv3, InceptionResNetv2, SqueezeNet, DenseNet201, MobileNetv2, ShuffleNet, Xception, NasNetmobile and Nasnetlarge] were utilised as a whole in generation of our AI models, and the transfer learning was used to optimise the fitness of the data. A specific AI model was established by training 80% of total samples with 10% of them used for model validation and the remaining 10% of the samples for testing diagnostic accuracy of the established model. (G) JAK2V617F causes morphological changes that allow distinguishing LSCs and other bone marrow leukaemic lineages from their normal cell counterparts.

Another issue we paid much attention to in building AI deep learning models was the G&W staining of cells. We realised that staining helps to visualise the cells, but the introduced colour signals are not relevant to cell morphology, thereby adding unwanted features that could be extracted by AI. To reduce the experimental variations derived from the cell staining, we turned the colour images of the cells to grey and enhanced grey images to create an image datastore prior to generating AI models (Figure 2A–E). This colour conversion also helps to simplify algorithms, eliminating the complexities related to deep learning requirements. The single‐cell images converted from G&W‐stained cells to grey colour cells in the image datastore were analysed by the 19‐CNNs pretrained using the ImageNet database to train AI deep learning models for distinguishing LSCs from their normal stem cell counterparts (Figure 2F). We conducted a binary analysis comparing JAKV617F‐expressing and non‐JAKV617F‐expressing subpopulations of LSK cells (LT‐HSCs, ST‐HSCs and MPPs). We used 80% of each subpopulation group of cell images to build an AI model and validated the model by reading the remaining 20% of the cell images that were unseen when the model was built. The classification accuracy was determined by binary analysis of the unseen images using the AI model built from 80% of the images. We observed that the classification accuracy for distinguishing JAK2V617F‐expressing LT‐HSCs from normal LT‐HSCs was greater than 90% (Figure 2G), indicating that the oncogenic JAK2V617F gene causes morphological changes that can be detected by our AI model for identifying LSCs in PV and separating them from their normal stem cell counterparts. Furthermore, JAK2V671F also caused morphological changes in ST‐HSCs and MPPs, allowing for distinguishing them from their normal cell counterparts (Figure 2G).

### 3.2 LSCs Are Morphologically Distinguishable From Other Bone Marrow Leukaemia Cells

In patients with PV or any other form of stem cell‐derived haematologic malignancies, LSCs mostly reside in bone marrow along with other malignant cells in different lineages, as well as normal bone marrow cells. We wondered whether we could specifically identify LSCs among all other types of malignant and normal bone marrow cells in PV mice to provide a conceptual basis for identifying and quantifying LSCs in future clinical practice. We showed that JAK2V617F‐expressing LT‐HSCs acquired unique morphological features that could be extracted and identified using our AI model (Figure 1). A key question to ask is whether we can use AI deep learning to identify LSCs and distinguish them from all other populations of cells (malignant or normal). To answer this question, we need to test whether we can identify JAK2V617F‐expressing LT‐HSCs from the entire bone marrow cell population that includes different lineages of malignant and normal haematopoietic cells. In this regard, we obtained each lineage of JAK2‐V617F‐expressing bone marrow cells from JAK2V617F knock‐in mice and normal bone marrow cells from WT mice by flow sorting. Single‐cell images for each lineage of the cells were collected for further analysis. We first mixed the single‐cell images of JAK2V617F‐expressing LT‐HSCs (LT‐HSCs Jak‐Cre) with all other types of JAK2V617F‐expressing cells (ST‐HSCs Jak‐Cre, MPPs Jak‐Cre, Lin^−^ Jak‐Cre and Lin^+^ Jak‐Cre) to train an AI model using 80% of the single‐cell images, and then used the remaining 20% of the images to test the AI model for classification accuracy. We found that JAK2V617F‐expressing LT‐HSCs were identified from the entire population of JAK2V617F‐expressing bone marrow cells by our AI model with about 97% accuracy (Table 1), indicating that LSCs in PV can be specifically identified and quantified using AI deep learning. Because various lineages of normal bone marrow cells would also be present in a PV patient, we decided to test whether we could develop an AI model to identify and quantify JAK2V617F‐expressing LT‐HSCs among all types of haematopoietic cells (malignant and normal). Therefore, we mixed the single‐cell images of JAK2V617F‐expressing LT‐HSCs (LT‐HSCs Jak‐Cre) with all other types of JAK2V617F‐expressing and normal cells (ST‐HSCs Jak‐Cre, MPPs Jak‐Cre, Lin^−^ Jak‐Cre, Lin^+^ Jak‐Cre, LT‐HSCs WT, ST‐HSCs WT, MPPs WT, Lin^−^ WT and Lin^+^ WT) to train and then test the AI model for classification accuracy. About 92% of classification accuracy was achieved (Table 1), demonstrating that we can use AI deep learning to identify and quantify LSCs in the mixture of all malignant and normal cell lineages.

**TABLE 1:** Identification of LSCs from total bone marrow cells in PV mice.

	Comparison groups	
	LT‐HSCs Jak‐Cre vs ST‐HSCs Jak Cre + MPPs Jak Cre + Lin^−^ Jak‐Cre + Lin^+^ Jak‐Cre	LT‐HSCs Jak‐Cre vs ST‐HSCs Jak Cre + MPPs Jak Cre + Lin^−^ Jak‐Cre + Lin^+^ Jak‐Cre + LT‐HSCs WT + ST‐HSCs WT + Lin^−^ WT + Lin^+^ WT
No. of cell images (training)	1080 vs 4860	1080 vs 11,925
No. of cell images (testing)	120 vs 540	120 vs 1205
Classification accuracy (%)	97.1	91.6

### 3.3 Multiple‐CNN Approach Is More Superior Than Single‐CNN Approach

**FIGURE 3:**
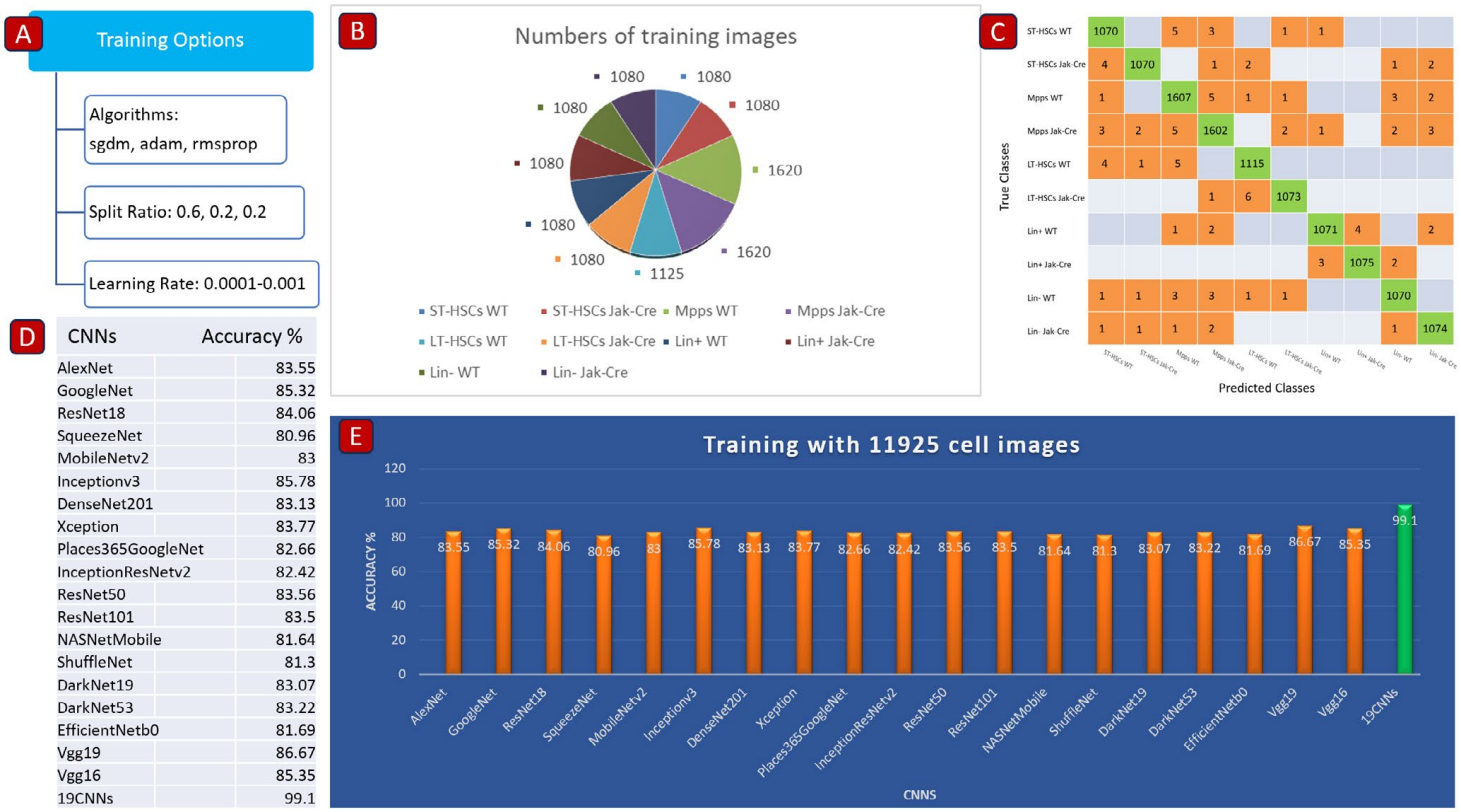
Enhanced performance with the multiple CNN approach. (A) Training options of AI models. (B) Numbers of 10‐types of single‐cell images to be analysed as a whole. (C) The confusion charts showing the numbers of the images that were correctly or incorrectly identified by the AI model using 19‐CNNs. (D‐E) Accuracy comparison between a single‐CNN approach and multiple‐CNN (19‐CNN) approach in identifying JAK2V617F‐expressing and non‐JAK2V617F‐expressing (LT‐SHCs, ST‐SHCs, MPPs and Lin^−^) cells.

We took a multiple‐CNN approach (19‐CNNs) in building AI models for distinguishing LSCs from other lineages of malignant cells in PV (Figure 1), because our previous work suggests that the multiple CNN approach led to achieving a higher classification accuracy than the single CNN approach did [6, 7]. Here, we intended to test directly whether the multiple CNN approach is more superior to the single CNN approach in classifying various lineages of JAK2V617F‐expressing and normal bone marrow cells from JAK2V617F knock‐in and WT mice. To train AI models (Figure 3A), we combined the single‐cell images of all 10 types of JAK2V617F‐expressing and normal bone marrow cells (LT‐HSCs Jak‐Cre, ST‐HSCs Jak‐Cre, MPPs Jak‐Cre, Lin^−^ Jak‐Cre, Lin^+^ Jak‐Cre, LT‐HSCs WT, ST‐HSCs WT, MPPs WT, Lin^−^ WT and Lin^+^ WT) to establish AI models using each CNN individually or 19‐CNNs as a whole, and then analysed 10 types of single‐cell images together (Figure 3B). The confusion chart showed the numbers of the images that were correctly or incorrectly identified by the AI model using 19‐CNNs (Figure 3C). We found that the combined 19‐CNN approach allowed for achieving an overall classification accuracy of 99.1%, whereas the single‐CNN approach reached a significantly lower classification accuracy (about 80.9%–86.6%) (Figure 3D,E). These results demonstrate that the use of multiple CNNs is more superior to that of any single CNN in building an AI model in classifying blood cancer cells in the bone marrow of PV mice.

## 4 Discussion

There have been no published methods allowing for visualising LSCs in haematologic malignancies microscopically, simply because unique morphological features cannot be extracted and identified by visual observation of these cells. In this study, we used AI deep learning to recognise the morphological differences among all lineages of malignant and normal cells, providing the first example for identifying and quantifying LSCs specifically by AI. Although the use of cell surface markers allows for recognising various lineages of haematopoietic cells by flow cytometry and other means, these methods do not distinguish between malignant and normal cell populations. We believe that there are unique morphological features in LSCs as well as other malignant cells and show, in fact, that AI deep learning allows us to distinguish these cells based on their morphological differences in PV mice. Our results promote us to propose a concept of AI Morphology, directing future research in identifying and quantifying LSCs and bulk of tumour cells not only in haematologic malignancies but also in other stem cell‐derived cancers. On the other hand, as mentioned above, cell surface markers do not distinguish between malignant and normal cell populations, but these markers could be combined with our AI approach to help to validate the detection of LSCs by AI.

There are a few key factors that ensure the success of our study. First, we carried out image processing to convert colour images to grey images that were processed by contrast enhancement functions. This helps to simplify algorithms and eliminate the complexities related to deep learning requirements. Second, transfer learning was employed to reduce the need for a large number of images and to speed up the training process by transferring knowledge from a previous process and then training it using relatively small datasets for the current task.

In current AI‐related research, a single CNN is often used. We provided evidence showing that our multiple CNN approach is superior to the single‐CNN approach in building AI models for analysing cell images of malignant and normal haematopoietic cells in the bone marrow of PV mice. To get multiple models to work together, we are not able to simply develop a model concatenation at the tail end of their layers because transfer learning requires every single CNN to remain independent. The aggregation of votes is an approachable way to combine 19‐CNNs into an executive model for classification. Our algorithm is to use a simple voting method with a majority rule. To classify a single image, the final classified category is decided by this majority rule. The majority rule is a decision rule that selects alternatives that have a majority: maximum votes among those models involved.

We need to point out that our use of 19‐CNN as a whole in building AI models and analysing cell images only requires a regular computer, which would be practical and convenient for future clinical use. On the other hand, we experienced that in the single‐CNN approach, which has been taken by almost all other research teams, it would be difficult to know or choose which particular CNN to use to achieve the highest accuracy without testing all CNNs individually; one‐by‐one testing of all CNNs is time‐consuming and expensive. By contrast, we have known that our multiple‐CNN approach (combined 19‐CNNs in this study) ensures the building of AI models that achieve the highest classification accuracy. In other words, our combined 19‐CNN approach ensures the best performance in analysing single‐cell images compared to the use of any single CNN.

Technically, We have known that variations of the images from different sources are often significant, relating to the types of the imaging machine/device, settings of the imaging machine/device, procedural differences in collecting the images, etc. This issue will raise a major challenge in clinical settings. One way to overcome this difficulty is to standardise the method for collecting any types of cell/tissue images across hospitals. Realistically, the standardisation requires huge efforts from the entire scientific community, which is difficult to do, and we plan to use a different strategy. If we used an AI model built by training cell images obtained from one source to analyse external, unfamiliar images from another source, we would combine a smaller set of the unfamiliar images into our prior training dataset to create a new training dataset and then re‐train an AI model by reading the combined images. As a result, the new AI model will be applicable to analysing future external cell images that are unfamiliar to the new AI model built from reading the combined images derived from both sources. We believe that this strategy is practical for the future use of our AI models in clinical settings. Another potential challenge in the clinic is the accuracy of disease diagnosis because it is not uncommon for pathologists to have different opinions when making diagnostic decisions on the same cases due to the complexity of some malignant diseases, which would impact how our AI models are built. To overcome this problem, we need to consider not only pathological diagnosis but also other clinical findings on a patient to confirm the type of disease this patient has, which will minimise errors in disease diagnosis.

## Author Contributions

**Dongguang Li:** conceptualization (equal), data curation (equal), formal analysis (equal), investigation (equal), methodology (equal), resources (equal), software (equal), validation (equal), visualization (equal), writing – original draft (equal). **Ngoc DeSouza:** investigation (equal), validation (equal), visualization (equal). **Kathy Nguyen:** investigation (equal). **Shaoguang Li:** conceptualization (equal), data curation (equal), formal analysis (equal), funding acquisition (equal), investigation (equal), methodology (equal), project administration (equal), resources (equal), software (equal), supervision (equal), validation (equal), visualization (equal), writing – original draft (equal).

## Ethics Statement

The present study followed international, national and/or institutional guidelines for humane animal treatment that is approved by our Institutional Animal Care and Use Committee.

## Conflicts of Interest

The authors declare no conflicts of interest.

## Data Availability

Original data related to this work can be obtained by contacting Dr. Shaoguang Li (shaoguang.li@umassmed.edu).
